# High Throughput Sequencing to Detect Differences in Methanotrophic Methylococcaceae and Methylocystaceae in Surface Peat, Forest Soil, and *Sphagnum* Moss in Cranesville Swamp Preserve, West Virginia, USA

**DOI:** 10.3390/microorganisms3020113

**Published:** 2015-04-02

**Authors:** Evan Lau, Edward J. Nolan, Zachary W. Dillard, Ryan D. Dague, Amanda L. Semple, Wendi L. Wentzell

**Affiliations:** Department of Natural Sciences and Mathematics, West Liberty University, 208 University Drive, CUB#139, West Liberty, WV 26074, USA; E-Mails: enolan@westliberty.edu (E.J.N.); zwdillard@westliberty.edu (Z.W.D.); RDAGUE@westliberty.edu (R.D.D.); amandasemple1@gmail.com (A.L.S.); wlwentzell@westliberty.edu (W.L.W.)

**Keywords:** Methanotrophic bacteria, high throughput sequencing, Illumina^®^ MiSeq, multiplex sequencing, microbial ecology, upland forest soils, peatlands, surface peat, *Sphagnum* moss, Methylococcaceae, Methylocystaceae, Evenness, Shannon-Weiner Index, Nonmetric multidimensional scaling, biogeography

## Abstract

Northern temperate forest soils and *Sphagnum*-dominated peatlands are a major source and sink of methane. In these ecosystems, methane is mainly oxidized by aerobic methanotrophic bacteria, which are typically found in aerated forest soils, surface peat, and *Sphagnum* moss. We contrasted methanotrophic bacterial diversity and abundances from the (i) organic horizon of forest soil; (ii) surface peat; and (iii) submerged *Sphagnum* moss from Cranesville Swamp Preserve, West Virginia, using multiplex sequencing of bacterial 16S rRNA (V3 region) gene amplicons. From ~1 million reads, >50,000 unique OTUs (Operational Taxonomic Units), 29 and 34 unique sequences were detected in the Methylococcaceae and Methylocystaceae, respectively, and 24 potential methanotrophs in the Beijerinckiaceae were also identified. *Methylacidiphilum*-like methanotrophs were not detected. Proteobacterial methanotrophic bacteria constitute <2% of microbiota in these environments, with the Methylocystaceae one to two orders of magnitude more abundant than the Methylococcaceae in all environments sampled. The Methylococcaceae are also less diverse in forest soil compared to the other two habitats. Nonmetric multidimensional scaling analyses indicated that the majority of methanotrophs from the Methylococcaceae and Methylocystaceae tend to occur in one habitat only (peat or *Sphagnum* moss) or co-occurred in both *Sphagnum* moss and peat. This study provides insights into the structure of methanotrophic communities in relationship to habitat type, and suggests that peat and *Sphagnum* moss can influence methanotroph community structure and biogeography.

## 1. Introduction

Methane (CH_4_) is responsible for ~20% of the current greenhouse gas radiative forcing, and is the most important greenhouse gas after carbon dioxide (CO_2_) [1]. Temperate forests and *Sphagnum* moss-dominated northern peatlands are significant global sources and sinks of methane, where anaerobically-produced CH_4_ is consumed by aerobic methanotrophic bacteria, which oxidize CH_4_ to CO_2_ or incorporate the carbon into biomass [2,3,4,5]. Aerobic microbial methane oxidation in aerated soils is the only major biological sink of methane and is critical in the removal of CH_4_ from these ecosystems before its release into the atmosphere [4,6], removing up to 90% of the annual gross CH_4_ production in wetlands and drained soils, and in the case of the latter, some methanotrophs can remove methane at atmospheric levels [7,8,9,10,11,12,13,14,15]. Methanotrophic bacteria have been detected in temperate forest soils, peat (dead organic matter) and endophytically in the hyaline cells and on external surfaces of *Sphagnum* moss, the dominant flora in peatlands. The diversity and/or abundances of methanotrophs in these environments have previously been described separately [10,16,17,18,19,20,21,22,23,24]. In these environments, oxygen availability and/or changes in the water level can affect CH_4_ fluxes and potentially affect methanotroph diversity and abundances [25]. For example, in temperate forest soils, the highest methane oxidation rates have been found in the topmost, aerated soils, whereas in *Sphagnum*-dominated peatlands the highest methane oxidation rates have been found in aerobic surface peat and mosses collected from near the water table [2,10,20,26]. However, to the best of our knowledge, no study has compared methanotroph diversity and abundances between these different environments with high methane oxidation rates, which are in close proximity to each other, in *Sphagnum*-dominated peatlands and neighboring temperate forest soils. Given the critical role of aerobic methanotrophs in reducing atmospheric methane flux, it is therefore imperative to evaluate and contrast their community structure in these locations to determine whether these habitats can influence their methanotrophic microbiota.

The vast majority of known aerobic methanotrophic bacteria are placed in the Proteobacteria, in the classes Alphaproteobacteria and Gammaproteobacteria, with a few members placed in the phylum Verrucomicrobia [23,27]. Based on 16S rRNA gene phylogeny, alphaproteobacterial methanotrophs are placed in the families Methylocystaceae, which includes the genera *Methylocystis* and *Methylosinus*, and Beijerinckiaceae, which includes the genera *Methylocapsa*, *Methylocella*, and *Methyloferula*. Meanwhile, gammaproteobacterial methanotrophs are placed in the family Methylococcaceae and Methylothermaceae, which include the genera *Methylococcus*, *Methylocaldum*, *Methylobacter*, *Methylomicrobium*, *Methylomonas*, *Methylosarcina*, *Methylosoma*, *Crenothrix*, *Clonothrix*, and *Methylosphaera* in the Methylococcaceae and *Methylohalobius*, *Methylothermus*, and *Methylomarinovum* in the Methylothermaceae [27,28]. While the Methylocystaceae and Methylococcaceae form coherent, monophyletic clusters in the Alphaproteobacteria and Gammaproteobacteria, respectively [2,23,27], the methanotrophic Beijerinckiaceae do not form a monophyletic cluster in the Alphaproteobacteria [29,30]. In the bacterial phylum Verrucomicrobia, members of the extreme acidophilic and methanotrophic genus, *Methylacidiphilum*, are placed in the family Methylacidiphilaceae [27,31].

Functional gene sequences for enzymes found in metabolic pathways have enabled the use of targeted PCR primers for gene amplification to describe microbial diversity. In the case of methanotrophs, however, this can result in the underestimation of methanotroph diversity. This is due to several cultivated methanotrophic taxa lacking the *pmoA* or *mmoX* genes, encoding a subunit of the particulate methane monooxygenase (pMMO) and a subunit of the soluble methane monooxygenase (sMMO), respectively, which are responsible for the initial step of methane oxidation to methanol [18,27,29,32]. In contrast, the nonfunctional 16S rRNA gene is ubiquitous, does not have this limitation, and, hence, may be better for assessment of aerobic methanotroph diversity in natural environments. Through the use of barcoded PCR primers targeting the V3 region of microbial 16S rRNA gene, the Illumina^®^ MiSeq multiplex NGS platform has high depth coverage of microbial diversity and can detect both abundant and rare microbes in diverse environments [33,34,35,36]. NGS (Next-Generation Sequencing) technology allows the assessment of microbiota both qualitatively (to determine diversity) and quantitatively (to determine abundance based on the frequency of sequences detected) to reveal and contrast microbial communities between habitats. However, it is critical in NGS studies of microorganisms to be aware of its weaknesses and limitations, including PCR biases, Taq polymerase fidelity, and 16S rRNA gene copy number variations between microorganisms and sequencing errors [37,38,39,40,41], which can potentially lead to errors in estimating diversity and sequence abundance. Nevertheless, these biases and errors can be minimized. For example, sequencing errors can be reduced by using fewer PCR amplification cycles [40], and within-sample heterogeneity can be reduced by using high DNA template concentrations during PCR [41].

To the best of our knowledge, NGS has not been used to compare the diversity and abundances between groups of methanotrophic bacterial communities (e.g., members of the Methylococcaceae *versus* members of the Methylocystaceae) within one habitat and between neighboring habitats within peatland and forest soil ecosystems. Knowledge from comparisons of methanotroph community structures based on 16S rRNA diversity patterns, particularly in combination with genomic data, may identify habitat-specific adaptations in methanotroph physiology and evolution. It can also reveal whether methanotrophic taxa tend to be detected only in one habitat or co-occur in different habitats, and consequently, this has implications for their biogeography and ecology [42]. In this study, barcoded universal PCR primers targeting the bacterial V3 region of the 16S rRNA gene were employed in Illumina MiSeq high throughput multiplex sequencing, to first create a bacterial gene library from sites undergoing high rates of methane oxidation: (i) the organic horizon of forest soils; (ii) aerated acidic surface peat; and (iii) submerged living *Sphagnum* moss tissues. Subsequently, sequences related to methanotroph families (*i.e.*, the Methylococcaceae, Methylocystaceae, and Beijerinckiaceae) were identified and tallied to assess their diversity and relative abundance according to habitat from amongst the sequence dataset of ~1 million reads. We hypothesized that these habitats harbored unique methanotroph assemblages, with distinct methanotrophic taxa occurring between all three habitats.

## 2. Experimental Section

### 2.1. Site Description, pH Measurement and Collections

Cranesville Swamp Preserve is a 1600 acre (650 ha) preserve situated in Preston County, West Virginia, and Garrett County, Maryland (39°31′53″N 79°28′55″W), and includes a 600 acre (243 ha) acidic *Sphagnum*-dominated bog (elevation 2560 ft. (780 m) above sea-level). The bog supports a diverse plant assemblage, including species of *Carex* sp., *Drosera* sp., *Eriophorum* sp., *Rhododendron* sp., *Sarracenia* sp., *Symplocarpus* sp., and *Vaccinium* sp. The bog is surrounded by a *Pinus*-dominated upland forest, which also includes trees from *Picea* sp., *Acer* sp., *Tsuga* sp., and *Betula* sp. Cranesville Swamp Preserve (39°31′53″N 79°28′55″W) is owned by the Nature Conservancy [43,44]. Water pH was measured in the field by means of a Fisher Science Education™ Water Quality pH Pen (Thermo Fisher Scientific Inc., Waltham, MA, USA) and soil pH was measured by means of Fisher Scientific Accumet AR20 pH/Conductivity Meter (Thermo Fisher Scientific Inc., Waltham, MA, USA). Samples for DNA extraction were collected on 15 May, 2012, from three distinct habitats for DNA extraction: (a) three samples from the topmost organic horizon of pine forest soil at the Cranesville Swamp Preserve trail marker 4, each weighing ~8 g; (b) three samples of acidic surface peat (at the air-water interface), each weighing ~5 g; and (c) three submerged *Sphagnum* moss thalli (near the air-water interface) weighing ~3 g each, from the same location off the boardwalk in the middle of Cranesville Bog. The sites for *Sphagnum* moss and peat collection were <20 cm from each other, and both sites are ~150 m from where the forest soil samples were collected.

### 2.2. DNA Extraction and Purification

DNA was extracted from soils using FastDNA™ SPIN kits (for soil) via bead beating (MP Biochemicals Inc., Santa Ana, CA, USA). Approximately 10–12 g of triplicate soil and peat samples, and 3 g of three *Sphagnum* moss samples were placed in multiple Multimix 2 tissue matrix tubes and processed in FastPreps at speed 5.5 for 30 s following the manufacturer’s instructions. DNA was then pooled into one tube for each sample—peat, soil, and *Sphagnum* moss. The DNA was further purified following the protocol described in Lau *et al.* (2007) [17]: briefly, extracted DNA was run on low-melting point (LMP) agarose gel, and stained gel bands of proper size were excised. Following the addition of ammonium acetate and unbuffered phenol, gel cutouts were melted in TE and DNA was re-extracted by centrifugation after the aqueous layer was precipitated by 95% ethanol. The community DNA yield ranged from ~50 to 280 ng/μL between the forest soil, peat and *Sphagnum* moss habitats.

### 2.3. Illumina Library Generation

The V3 region of the 16S rRNA gene was PCR-amplified using modified universal bacterial 341F and 518R primers [45] complementary to Illumina forward, reverse, and multiplex sequencing primers (with the reverse primer also containing a 6-bp index allowing for multiplexing) following the complete protocol described in Bartram *et al.* (2011) [33]. The reverse primers were appended with sample-specific barcodes, adapted from Bartram *et al.* (2011) [33] for PCR reactions: “V3_F modified,” as forward primer for all 3 reactions, and barcoded reverse primers “V3_1R” for soil DNA, “V3_2R” for peat, and “V3_3R” for *Sphagnum* moss. The primers were synthesized salt-free by Eurofins MWG Operon (Eurofins Genomics, Huntsville, AL, USA). For DNA from each habitat, three separate PCR amplifications in 50 μL reaction mixtures were carried out using BioReagents™ exACTGene™ Complete PCR Kit and Core Reagent Sets (Thermo Fisher Scientific Inc., Waltham, MA, USA) at 24 PCR cycles, for a total of 9 reactions. Separation of products from primers and primer dimers was accomplished by electrophoresis on a 2% LMP agarose gel, and PCR products of the correct size were band-excised and recovered using a QIAquick gel extraction kit (Qiagen, Venlo, Limburg, Netherlands). For each library, triplicate soil PCR products with unique indexes (from the reverse primers) were combined and quantified on a NanoDrop UV-Vis ND2000 spectrophotometer (Thermo Fisher Scientific Inc., Waltham, MA, USA), and sent to West Virginia University Genomics Core Facility [46] for high throughput multiplex sequencing. DNA concentration from all three samples were analyzed on an Agilent 2100 Bioanalyzer System (Agilent Technologies, Inc., Santa Clara, CA, USA) and subsequently verified with Biorad IQ5 real time thermocycler (Bio-Rad Laboratories, Inc., Hercules, CA, USA) using Illumina quantification standards from KAPA Biosciences (KAPA Biosystems, Wilmington, MA, USA) as per manufacturer’s protocol. The samples were then sequenced on an Illumina^®^ MiSeq [47] using 150-nucleotide paired-end multiplex sequencing (Illumina, San Diego, CA, USA). The library was sequenced in a single channel of a flow cell using v2 sequencing reagents and protocol, generating paired reads of ~190 bases.

### 2.4. Bioinformatic Analyses

Processing of the raw sequence data was initially performed in Basespace using Illumina^®^ metagenomic pipeline [48]. Merging the paired reads and further analyses were carried out using Axiome with installed PANDAseq and the Quantitative Insights Into Microbial Ecology (QIIME v.1.8.0) [49] pipeline on the Linux platform, at default settings unless otherwise stated, including taxaplot for overall bacterial diversity, and calculations of (i) Chao1 to estimate taxon richness; and (ii) rarefaction curves to calculate species richness for a given number of individual sequences sampled [50,51,52]. On Pandaseq, the minimum overlap length was set at the threshold of 0.9. The read length maximum for all sequences was 195 bp, but ranged, as follows, for sequences related to the following families: Methylocystaceae and Beijerinckiaceae (169 bp), and the Methylococcaceae (193 bp). Since the V3 region of the 16S rRNA gene is conserved, reads were removed from further analysis if any one of the following criteria was met: (i) reads were ≥2 bp shorter than the maxima mentioned above; (ii) the number of ambiguous bases was ≥1; and (iii) presence of homopolymers with >4 bp. The clustering of the sequences into operational taxonomic units (OTUs) was initially performed using UCLUST [53,54] and default cutoff value of 97% sequence identity. The taxonomic identity of a representative sequence from each cluster was classified using the RDP [55,56] classifier in Pandaseq. The confidence threshold was set at 60% (below the default cutoff value of 80%) in order to retrieve as many potential sequences from methanotrophic taxa as possible from the Methylococcaceae, Methylocystaceae, and Beijerinckiaceae.

### 2.5. Phylogenetic Analyses

Phylogenetic trees were constructed using MEGA 6.06 [57]. 16S rRNA gene sequences of known representative methanotrophs from the Methylococcaceae, Methylocystaceae, and Beijerinckiaceae were downloaded from GenBank [58], in all phylogenetic analyses. Gene sequences were aligned using the MUSCLE algorithm in MEGA 6.06 and manually inspected. The final alignments consisted of 193, 169, and 169 characters, comprising 71 taxa, 70 taxa, and 66 taxa, respectively, for sequences related to the Methylococcaceae, Methylocystaceae, and Beijerinckiaceae. Phylogenetic reconstruction was implemented using Maximum Parsimony (MP), with complete deletion if gaps were present, and tree-bisection-reconnection (TBR) utilized for tree generation. The resulting trees were obtained via random stepwise addition of sequences, at MP search level 3, with 25 initial trees generated from a heuristic search. Unless stated otherwise, statistical support for all trees was obtained from 1000 bootstrap replicates under the same initial settings (only bootstrap values ≥50% are reported). To verify that 16S rRNA gene sequences placed in the Methylococcaceae and Methylocystaceae were indeed methanotrophic, sequences were removed if they share ≤95% identity with known methanotrophs and the resulting phylogenetic trees showed ≤80% bootstrap support for the monophyletic Methylococcaceae and Methylocystaceae. Additional rarefaction curves for these putative methanotrophs (in addition to those already generated for microbial diversity) were implemented using the VEGAN package in R [59], to determine methanotrophic species richness for the number of sequences sampled.

Pairwise base comparisons of 16S rRNA nucleotide sequences between OTUs and their closest relatives were determined using BLAST [60]. Pairwise distance was calculated by MEGA 6.06 and reported as % identity values.

### 2.6. Statistical and Ecological Analyses

To count the abundance (*i.e.*, frequency) of each methanotroph OTU, a proprietary program was written in the open-source Python language (v.2.7) [61] to iterate through the file for those selected methanotroph OTUs, which were verified upon phylogenetic analyses described above. The sequence tags were then used as an indicator of location (0, 1, or 2, where 0 = peat, 1 = soil, 2 = *Sphagnum* moss) and summed accordingly, per OTU. The OTU abundance results were summarized in a table containing the information on taxonomic affiliation, closest known cultured methanotrophic relative(s) and the number of reads assigned to each OTU at each habitat (peat, soil, and *Sphagnum* moss).

Methanotroph diversity was compared between the habitats where each methanotroph OTU occurred (i) singly (in one habitat only); (ii) in two habitats; or (iii) across all three habitats. Shannon-Weiner Index (H) for each site was calculated using the equation: H = −sum (P_i_ ln [P_i_]), where P_i_ is the proportion of individuals belonging to the *i*th species in the dataset. Species evenness (E) was calculated using the equation: E = H/ln(S), where H is Shannon-Weiner Index, and S is the total number of species. Chi-square distance matrices were created from the occurrence frequencies within the IBM Statistical Package for the Social Sciences (SPSS), ver. 20, for Windows (IBM Corp., Armonk, New York, NY, USA). Nonmetric Multidimensional Scaling (NMDS) was performed with the distance matrices from methanotroph OTUs using the ALSCAL algorithm in SPSS, set to two dimensions, with untying of tied observations. NMDS analyses were set for 60 iterations (unless results for subsequent analyses were identical) to ensure the lowest stress values. Results from NMDS of OTUs from the Methylococcaceae and Methylocystaceae were presented in separate two-dimensional ordination plots.

### 2.7. Nucleotide Sequence Accession Numbers

All partial 16S rRNA gene sequence data are available through the European Bioinformatics Institute (EBI) with project accession number PRJEB7335. The nucleotide sequences for the partial 16S rRNA genes have been deposited in EBI under individual accession nos. LN624830–LN62527, and LN649237–LN649239.

## 3. Results

### 3.1. pH Measurement

pH of bog water at the sites of collection was measured at 5.3–5.4. pH of collected soil was measured at 3.6.

### 3.2. Bacterial Taxon Richness and Diversity

Partial 16S rRNA gene sequences were retrieved from (i) aerated acidic surface peat, (ii) submerged *Sphagnum* moss tissues, and (iii) organic horizon of forest soils. The 991,308 quality reads from raw data, of which 134,868, 537,990 and 318,450 reads originated from surface peat, forest soil, and *Sphagnum* moss, respectively, underwent quality filtration and denoising to produce a total of 52,134 unique OTUs with read lengths ranging from ~164 bp to 195 bp. There were 5107 sequences that could not be assigned to any particular phylum. Significant proportions of Acidobacteria and Proteobacteria were detected consistently at >20% of the total composition at all three sites. A summary of the bacterial phyla in which the 52,134 unique sequences are placed for surface peat, forest soil, and *Sphagnum* moss is shown in Figure S1. Our multiplex sequencing approach covered the following percentages of observed species-level richness (in comparison to Chao1, the estimated richness) for the following samples: 52.3% (surface peat), 61.3% (forest soil), and 59.7% (*Sphagnum* moss). Rarefaction curves, which assess bacterial taxon richness in surface peat, forest soil, and submerged *Sphagnum* moss, are shown in Figure S2a.

### 3.3. Phylogeny of 16S rRNA Sequences Related to Methanotrophs

A total of 29 unique OTUs related to the Methylococcaceae, 34 unique OTUs related to the Methylocystaceae, and 24 unique OTUs related to the Beijerinckiaceae were identified for further phylogenetic and statistical analyses. Based on abundance data, these OTUs constitute <1% of total bacterial abundance. The rarefaction curves of methanotroph OTUs (from the Methylococcaceae and Methylocystaceae) identified above, which were analyzed separately, are shown in Figure S2b.

**Figure 1:**
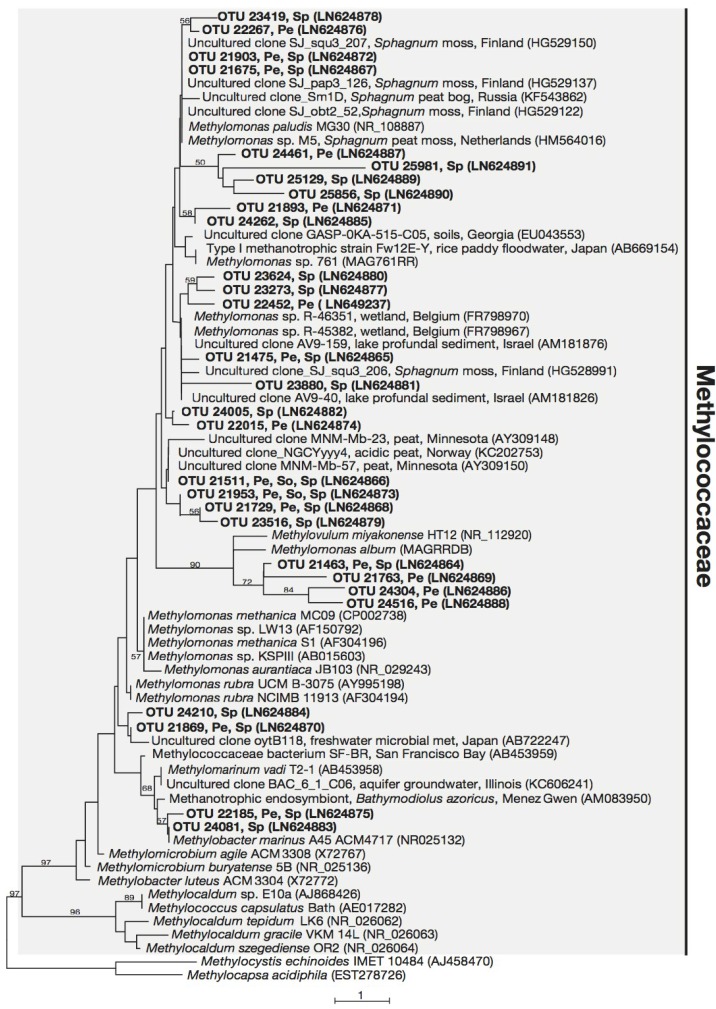
Phylogenetic tree based on maximum parsimony (MP) analysis of twenty-nine OTUs (~193 bp) detected in this study (in bold) in comparison with their close relatives and representatives from the Methylococcaceae. The locations where these OTUs were detected are indicated: Pe, peat; So, forest soil; Sp, *Sphagnum* moss. Bootstrap values from 1000 replicates are indicated at the nodes of branches (if > 50). The scale bar represents the number of nucleotide changes.

**Figure 2:**
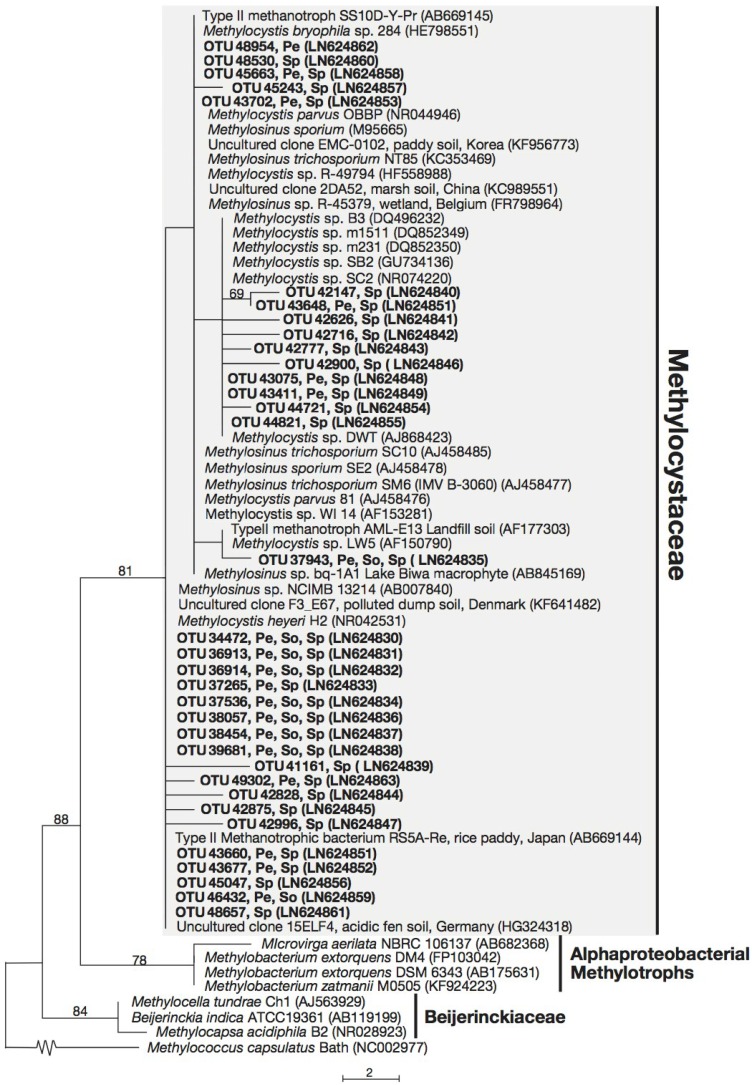
Phylogenetic tree based on maximum parsimony (MP) analysis of thirty-four OTUs (~169 bp) detected in this study (in bold) in comparison with their close relatives and representatives from the Methylocystaceae. The locations where these OTUs were detected are indicated: Pe, peat; So, forest soil; Sp, *Sphagnum* moss. Bootstrap values from 1000 replicates are indicated at the nodes of branches (if > 50). The scale bar represents the number of nucleotide changes.

**Figure 3:**
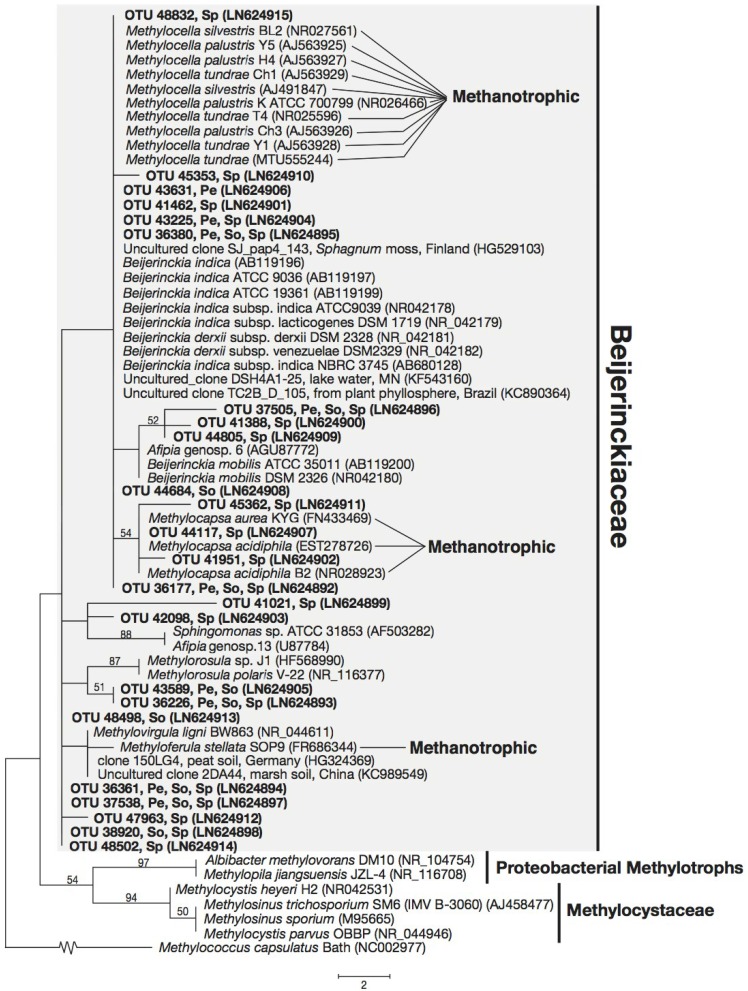
Phylogenetic tree based on maximum parsimony (MP) analysis of twenty-four OTUs (~169 bp) detected in this study (in bold) in comparison with their close relatives and methanotrophic representatives from the Beijerinckiaceae. The locations where these OTUs were detected are indicated: Pe, peat; So, forest soil; Sp, *Sphagnum* moss. Bootstrap values from 1000 replicates are indicated at the nodes of branches (if > 50). The scale bar represents the number of nucleotide changes.

All 29 OTUs related to the Methylococcaceae were placed in a monophyletic cluster which include cultured members of the Methylococcaceae, with strong support (Figure 1), while the 34 unique sequences related to the Methylocystaceae fell within the monophyletic clade consisting of *Methylosinus* and *Methylocystis* species (Figure 2). The 24 unique sequences related to the Beijerinckiaceae fall within an unsupported cluster, which included both methanotrophic Beijerinckiaceae (from genera *Methylocella*, *Methylocapsa*, and *Methyloferula*) and non-methanotrophic Beijerinckiaceae (e.g., from genera *Beijerinckia*, *Afipia*, *Methylorosula* and *Methylovirgula*) (Figure 3).

Of the 29 OTUs detected and clustering within the Methylococcaceae, 24 OTUs (83% of these OTUs) were most closely-related to several *Methylomonas* sp. (with 95%–99% 16S rRNA sequence identity). The locations in which their closest relatives were isolated from or detected in span a range of environments, including peat, *Sphagnum* moss, rice paddy fields and marine habitats (Figure 1). Closest relatives to these 24 OTUs include *Methylomonas* sp. R-45382 isolated from a wetland [62] and *Methylomonas paludis* strain MG30 [63], an acid-tolerant methanotroph isolated from peat. OTU 21511 and OTU 21953 were the only two OTUs detected in forest soil (see Figure 1; Table S1). Both sequences are also detected in peat and *Sphagnum* moss. Two other sequences related to the Methylococcaceae, OTU 22185 and OTU 24081, detected in peat and *Sphagnum* moss, and *Sphagnum* moss only, respectively, are most closely-related to *Methylomarinum vadi* [64], a recently isolated marine methanotroph (with 96% 16S rRNA sequence identity), and are also closely-related to other aquatic methanotrophs, as well as *Methylobacter* spp.

For the 34 sequences placed in the Methylocystaceae, 50% were most closely-related to *Methylocystis* sp. (with 99%–100% identity). Of these, five sequences in the Methylocystaceae, namely, OTU 34472, OTU 36913, OTU 37526, OTU 36981, and OTU 42875 are most closely-related to *Methylocystis heyeri* strain H2 and *Methylocystis heyeri* strain Sak, isolated from acidic peat and acidic tropical forest soil, respectively [65] (see Figure 2; Table S1). In addition, 13 sequences are most closely-related (with 99%–100% sequence identity) to Type II methanotrophic strain RS5A-Re isolated from rice paddy soil [66]. Finally, 10 sequences detected in peat and *Sphagnum* moss or *Sphagnum* moss only, are most closely-related to *Methylocystis* sp. strains SC2 and SB2 (99%–100% sequence identity), which were isolated from peat and aquifer environments, respectively [67,68].

In the Beijerinckiaceae, the methanotrophic genera *Methylocapsa*, *Methylocella* and *Methyloferula* are placed polyphyletically and, thereby, do not share a coherent common evolutionary origin based on 16S rRNA gene phylogeny. This is in agreement with previous studies [23,30,69,70,71] (Figure 3). Here, three sequences, OTU 41951, OTU 44117, OTU 45362, with 98%–99% nucleotide sequence identity to methanotrophic *Methylocapsa aurea* strain KYG, isolated from forest soil [72], form a weakly-supported monophyletic cluster with all known members of the genus *Methylocapsa* (*i.e.*, *M. acidiphila*, *M. acidiphila* strain B2, *M. aurea* strain KYG). All three sequences are detected only in *Sphagnum* moss (see Figure 3; Table S2). Additionally, six sequences, OTU 36177, OTU 36380, OTU 41462, OTU 43631, OTU 45353, and OTU 48832 show 96%–100% sequence identity to *Methylocella silvestris* strain BL2, a methanotroph, and *Beijerinckia indica* subsp. *indica* strain ATCC 9039, a non-methanotroph. For the relatively short region of the 16S rRNA gene employed in this study (~169 nucleotides for proteobacterial sequences), we note that the nucleotide sequences of methanotrophs from genus *Methylocella* (*M. silvestris*, *M. tundrae*, *M. palustris*) share 100% identity with several strains of the non-methanotroph, *Beijerinckia indica*. It is possible some of the abovementioned OTUs belong to methanotrophs.

None of the OTUs were placed in the family Methylothermaceae, NC10 phylum [73], or the Methylacidiphilaceae [27] cluster. Only OTU 20595, detected only in *Sphagnum* moss, showed 89% sequence identity with the methanotroph *Methylacidiphilum infernorum* strain V4 [31] (see Figure S3).

### 3.4. Statistical and Ecological Analysis of Putative Methanotroph 16S rRNA Gene Sequences

Due to the uncertainty about the phylogeny of methanotrophs in the Beijerinckiaceae, statistical analyses were carried out only for environmental sequences placed in the Methylococcaceae and Methylocystaceae from this study.

**Figure 4:**
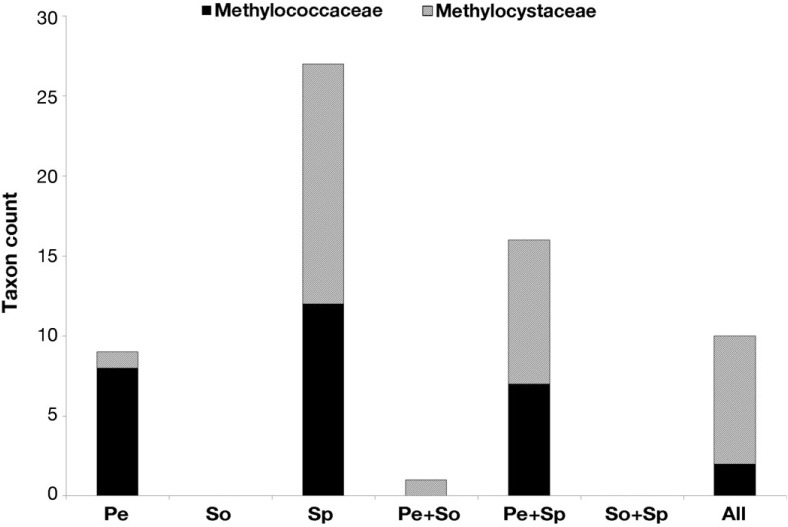
Graph of methanotroph 16S rRNA sequences related to the Methylococcaceae and Methylocystaceae and the habitats where they occurred. OTUs which only occurred (i) singly in one habitat only (peat, forest soil, and *Sphagnum* moss); (ii) in 2 habitats only (peat + forest soil, Peat + *Sphagnum* moss, and forest soil + *Sphagnum* moss) or (iii) across all three habitats. Most of these methanotrophs (~57% of the OTUs) detected in surface peat, forest soil and *Sphagnum* moss are unique and are not detected in another habitat. In contrast, only a small proportion of these methanotrophs (~15% of the OTUs) co-occur in all three habitats. Pe, peat; So, forest soil; Sp, *Sphagnum* moss; All, all habitats.

The 29 unique OTUs related to the Methylococcaceae were represented by a total of 136 reads, whereas the 34 OTUs related to the Methylocystaceae were much more abundant, represented by 4361 reads (see Table S1). Based on OTU abundance data, the Methylocystaceae comprise the vast majority of the methanotroph population, with the Methylococcaceae abundance at ~6%, ~0.6%, and ~2% that of the Methylocystaceae in peat, forest soil, and living *Sphagnum* moss, respectively (see Table S1). A total of 20 OTUs from the Methylococcaceae and 16 OTUs from the Methylocystaceae occurred singly, in peat only, and in living *Sphagnum* moss only, with 27 unique OTUs from both families occurring in *Sphagnum* moss (Figure 4). In contrast, seven OTUs from the Methylococcaceae and 10 OTUs from the Methylocystaceae occurred in two habitats, with the vast majority of them (16 out of 17) co-occurring in peat and *Sphagnum* moss. Only 10 OTUs (two from the Methylococcaceae, eight from the Methylocystaceae) co-occurred in all three habitats. Ten of the 11 sequences detected in soil were the same 10 OTUs which co-occur in all three habitats (Figure 4).

### 3.5. Shannon-Weiner Diversity Index (H) and Species Evenness (E)

**Figure 5:**
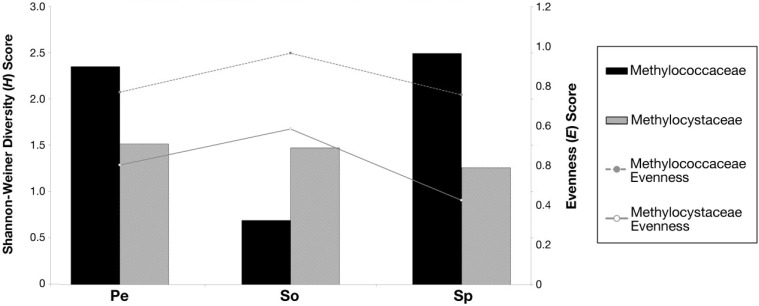
Calculated Shannon-Weiner (*H*) and evenness (*E*) indices for methanotroph 16S rRNAs related to the Methylococcaceae and Methylocystaceae for each habitat—surface peat, forest soil, and *Sphagnum* moss. Pe, peat; So, forest soil; Sp, *Sphagnum* moss.

The Shannon-Weiner diversity index (*H*) is directly proportional to the number of taxa and inversely proportional to the number of individuals for each taxon. In peat and *Sphagnum* moss, higher *H* values were observed for sequences related to the Methylococcaceae than for the Methylocystaceae (Figure 5). Since the number of sequences detected in peat and *Sphagnum* moss habitats that are placed in the Methylococcaceae and Methylocystaceae vary within an order of magnitude (see Table S1), this suggests that in peat and *Sphagnum* moss habitats, the sequences related to the Methylococcaceae are just as diverse as the Methylocystaceae, despite the Methylococcaceae being less abundant overall. The *H* value for the Methylococcaceae was lowest in forest soil, as only two OTUs from the Methylococcaceae were detected there. The evenness value (*E*) is inversely proportional to number of individuals per taxon, with a lower evenness value indicating more even proportions of taxa, and a higher value indicating uneven taxa distribution. Overall, the Methylococcaceae show higher evenness values (*i.e.*, are less even) than the Methylocystaceae for all three habitats (Figure 5).

### 3.6. NMDS Analysis of 16S rRNA Sequences Related to the Methylococcaceae and Methylocystaceae.

**Figure 6:**
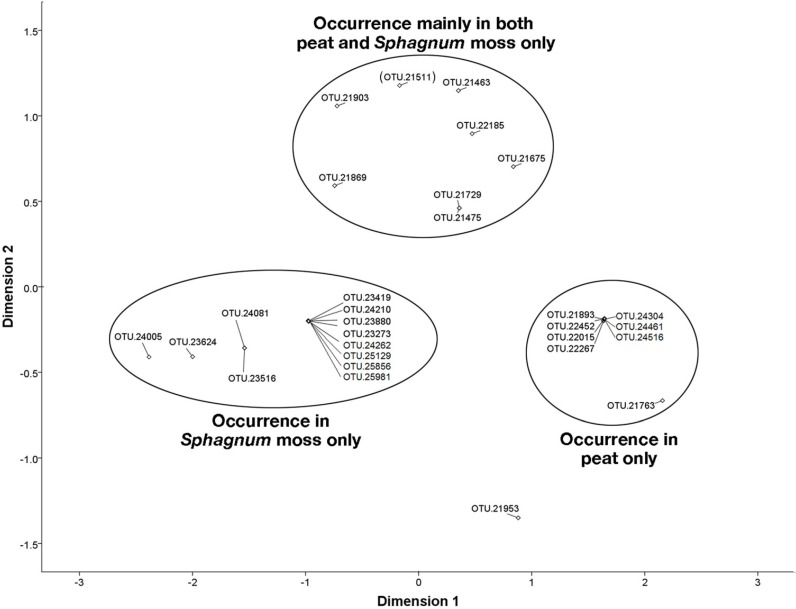
Similarity in the presence and abundance of the Methylococcaceae detected in peat, forest soil, and *Sphagnum* moss as a two-dimensional plot of nonmetric multidimensional scaling (NMDS; Stress = 0.07667, *R*^2^ = 0.97896). OTUs on the NMDS plot that are closer to one another are more similar in their occurrence. The OTUs from the Methylococcaceae occurring in peat only, *Sphagnum* moss only and mainly in peat and *Sphagnum* moss only, with the exception of OTU 21511 (in parenthesis), which also occurred in forest soil, in addition to peat, and *Sphagnum* moss, appear to be more similar to one another.

**Figure 7:**
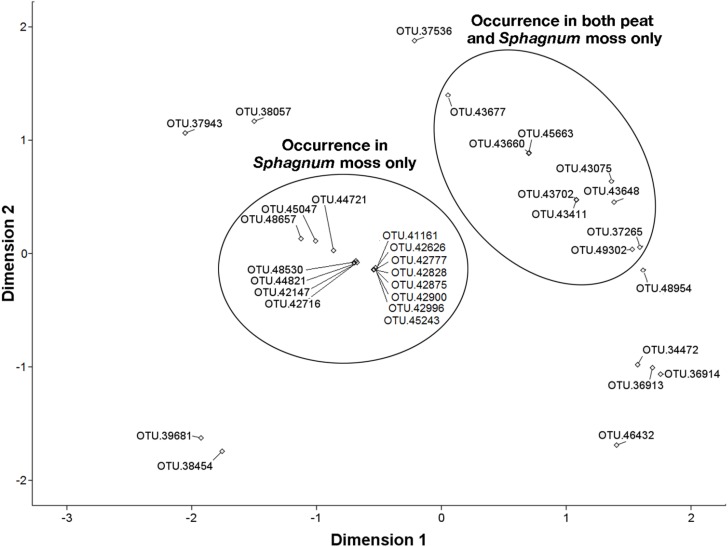
Similarity in the presence and abundance of the Methylocystaceae detected in peat, forest soil, and *Sphagnum* moss as a two-dimensional plot of nonmetric multidimensional scaling (NMDS; Stress = 0.18333, *R*^2^ = 0.87487). OTUs on the NMDS plot that are closer to one another are more similar in their occurrence. The OTUs from the Methylocystaceae occurring in *Sphagnum* moss only and in peat and *Sphagnum* moss only appear to be more similar to one another.

Analysis iterations for OTUs from the Methylococcaceae and Methylocystaceae were terminated at the 8th and 9th iterations, respectively, as the results of ranked distances between methanotroph OTUs were not significantly different thereafter. On the NMDS plots, proximity between OTUs corresponds to their similarity [74]. The OTUs from the Methylococcaceae (Stress = 0.077, *R*^2^ = 0.979) were distinctly separated into three clusters based on occurrence in habitats: *Sphagnum* moss only, peat only, and both peat and *Sphagnum* moss [with the exception of OTU 21511 (see Table S1) in the last cluster, which occurred in peat, forest soil, and *Sphagnum* moss] (Figure 6). From the Methylocystaceae, the OTUs detected in *Sphagnum* moss only, and both peat and *Sphagnum* moss were placed in separate clusters when visualized through NMDS (Stress = 0.183, R^2^ = 0.875) (Figure 7).

## 4. Discussion

Natural environments such as temperate forest soils and acidic boreal peatlands are complex, with phylogenetically heterogeneous microbial species potentially possessing overlapping metabolic abilities (*i.e.*, different populations performing similar functions). Aerobic proteobacterial methanotrophs are unique because their distinct monophyletic placement in some families (*i.e.*, the Methylococcaceae and Methylocystaceae) allows one to directly link phylogenetic and taxa abundance data to an important biogeochemical function—aerobic methane oxidation, the only major biological sink of methane in upland temperate forests and *Sphagnum* moss-dominated peatland ecosystems. Hence, describing the community structure of 16S rRNA gene sequences that cluster within those clades from different habitats within these ecosystems can contribute to our understanding of the critical roles these methanotrophs play in ecosystem functioning.

In this study, high throughput sequencing of 16S rRNA amplicons was conducted on DNA extracted from three different neighboring habitats in a nature preserve—surface peat, upland temperate forest soil and submerged, living *Sphagnum* moss. From nearly 1 million reads, we retrieved sequences related to methanotrophic taxa from the Methylococcaceae and Methylocystaceae, and potentially from the Beijerinckiaceae. The community structures of these putative methanotrophic bacteria were examined through both qualitative and quantitative 16S rRNA gene approaches. We detected 63 sequences related to the Methylococcaceae and Methylocystaceae from Cranesville Swamp Preserve, WV, USA, which constitute only <2% of all bacterial taxa detected and their abundances (based on frequencies of occurrence) were <1% of the total reads. The vast majority of OTUs from the Methylococcaceae and Methylocystaceae were closely-related to *Methylomonas* sp., *Methylovolum* sp. and *Methylocystis* sp., respectively, consistent with studies on methanotroph diversity in *Sphagnum* moss from a Dutch peat bog and wetlands of European Northern Russia [20,21,75]. Given the poorer resolving power of the V3 region of the 16S rRNA in discriminating between methanotrophs from the closely-related genera *Methylocella* and *Beijerinckia*, and the lack of monophyly amongst methanotrophs in the Beijerinckiaceae, it was not possible to predict the phenotype of any novel member in this family based on our 16S rRNA gene sequence information alone. Hence, the physiologies of these 24 OTUs which are related to the Beijerinckiaceae are unknown. It is believed that the methanotrophs likely responsible for methane oxidation at atmospheric levels in temperate forest, members of the USCα cluster, are closely related to *Methylocapsa acidiphila*, a member of the Beijerinckiaceae, based on *pmoA* gene-based phylogeny, which would not have been detected using our approach, as their phylogenetic placement based on the 16S rRNA gene is unknown [17,19]. None of the OTUs were closely-related to the family Methylacidiphiliaceae (≤89% sequence identity to the said family). Our results are in agreement with Serkebaeva *et al.* (2014) [24], which failed to detect the same verrucomicrobial methanotrophs in acidic surface peat in European North Russia. Due to the abovementioned reasons, only methanotrophic sequences related to the Methylococcaceae and Methylocystaceae were used for further analyses.

Our data, based on the abundance of individual OTUs, indicates there are ~16–170 times more Methylocystaceae compared to Methylococcaceae in forest soil, peat, and mosses. These observations are consistent with results from studies based on FISH (fluorescence *in-situ* hybridization) and methanotroph-specific gene clone libraries on the uppermost (organic) layer of upland forest soils, aerated peat, with porous hyaline cells and the latter and living *Sphagnum* moss, which show the Methylococcaceae constitute a small minority of the methanotrophic populations [11,17,75,76,77] but are contrary to two studies which indicate that (i) up to 50% of methanotrophs in the peat of a Dutch peatland belonged to the Methylococcaceae [20,21]; and (ii) no methanotrophs were detected in *Sphagnum* moss tissues in Austrian alpine bogs [78]. Even though the Methylococcaceae are far outnumbered by the Methylocystaceae in all habitats, they are nearly as diverse as the Methylocystaceae in peat and *Sphagnum* moss, but not in forest soil.

Within the peatland, living *Sphagnum* moss and non-living surface peat are different habitats for microorganisms, with the former being moss thalli with porous hyaline cells and the latter consisting of decomposing organic matter [16,20,77]. Our analyses indicate living *Sphagnum* moss harbor an assemblage of methanotrophs belonging to both the Methylococcaceae and Methylocystaceae (mainly from the genera *Methylomonas*, *Methylocystis* and *Methylosinus*), some of which are novel and unique to *Sphagnum* moss only and absent in the other two habitats. A significant number of methanotrophs (16 OTUs, seven from the Methylococcaceae and nine from the Methylocystaceae) also tend to co-occur in living *Sphagnum* moss and peat (but not in aerated forest soils), which are located <20 cm from each other in our field site, with the peat located at the air-water interface and the living *Sphagnum* moss was submerged just below the air-water interface. This suggests that some methanotrophs may either be ubiquitous in peat and living *Sphagnum* moss, or are able to move through the water between both habitats. Methanotrophs detected in forest soil tend to co-occur in the other two habitats, peat and *Sphagnum* moss, whereas the majority of OTUs detected in peat and *Sphagnum* moss (52 OTUs detected in both habitats) tend not to occur at all in forest soil (Table S1). This suggests that the methantrophs from forest soil (two OTUs from the Methylococcaceae and eight OTUs from the Methylocystaceae) are better adapted to surviving in different environments compared to those occurring in either peat only or *Sphagnum* moss only. Proteobacterial methanotroph diversity and NMDS suggest that certain habitat types (*i.e.*, peat only, *Sphagnum* moss only, peat and *Sphagnum* moss only, and all 3 habitats) tend to contain unique proteobacterial methanotroph communities (see Figure 4 and Table S1). This suggests that the habitat may influence the community structure of the methanotrophs in peatlands and northern temperate forest soils.

The results generally support our hypothesis, and over half (~57% of the 63 OTUs) of the methanotrophs from the Methylococacceae and Methylocystaceae in surface peat, forest soil, and *Sphagnum* moss are unique and are not detected in another habitat. On the contrary, only a small proportion of these methanotrophs (~15% of the OTUs) co-occur in all three habitats. These results also provide insights into the community structure of methanotrophs from the Methylococacceae and Methylocystaceae in these habitats. They suggest that habitat can select for, or influence methanotroph community structure and may affect methanotroph biogeography, as previously suggested by Lüke *et al.* (2010) [42] for paddy field methanotrophs. Additionally, unlike Lüke *et al.* (2010), the OTUs from the Methylococcaceae and Methylocystaceae, which occur in (i) peat only; (ii) *Sphagnum* moss only and (iii) both peat and *Sphagnum* moss, did not descend from one common ancestor in both families. However, some OTUs were placed within clusters consisting predominantly of taxa found or detected in wetlands, peat or *Sphagnum* moss worldwide.

In our study, instead of creating group-specific PCR primers based on the 16S rRNA gene for each family of methanotrophs, which may potentially introduce additional biases, we used a set of widely-used PCR primers [33,45] and we limited the number of PCR cycles to 24, while using high DNA template concentrations to reduce PCR bias and PCR errors in our Illumina-based multiplex sequencing to more comprehensively describe and compare methanotrophic populations between different sites. From an inspection of sequenced methanotroph genomes from the Methylococcaceae, Methylocystaceae and Methylacidiphilaceae, and methanotrophic Beijerinckiaceae on the databases [79,80], the copy number of methanotroph 16S rRNA gene(s) tend to range from 1 to 4 in general. While this variation is not abnormally high, it may potentially inflate the abundance numbers of methanotrophs relative to other microorganisms from our data.

We detected and described uncultured aerobic methanotrophs from triplicate samples of very different environments, peat, living *Sphagnum* moss and temperate forest soils. Based on the number of cultured methanotrophic genera, the Methylococcaceae and Methylocystaceae currently constitute the vast majority of known aerobic methane oxidizing bacteria. Hence, the assessment of the community structure and abundances of sequences belonging to both families can reveal critical information on the effect of a substantial portion of methanotrophs on their ecosystems. We show that habitat type, particularly surface peat and *Sphagnum* moss, despite being very close to one another, may influence the community structure of these methanotrophs and may affect their adaptations to their habitats in the aforementioned ecosystems. Our study included only samples collected from three representative sites undergoing high rates of methane oxidation. Further studies involving wider samplings, spatially, across peatlands (*i.e.*, surface and subsurface peat, bog water, submerged and non-submerged *Sphagnum* moss) and temperate forest soils at different depths may be required to verify our conclusions on methanotroph community structure and biogeography in temperate and boreal ecosystems.

## 5. Conclusions

In this study, PCR primers targeting universal Bacterial 16S rRNA genes were used in Illumina MiSeq multiplex sequencing to describe and compare methanotrophic populations from the proteobacterial families Methylococcaceae and Methylocystaceae between different habitats known to harbor diverse methanotrophic communities and undergo high rates of methane oxidation. The sites include the organic horizon of forest soil, surface peat, and submerged *Sphagnum* moss, from Cranesville Swamp Preserve, West Virginia. We were able to detect 63 OTUs from both families, even though they constitute <2% of microbiota in these environments. The majority of methanotrophs detected tend to occur in peat or *Sphagnum* moss or co-occurred in both *Sphagnum* moss and peat. This study provides insights into the community structure and biogeography of methanotrophic communities in relationship to habitats such as peat and *Sphagnum* moss within *Sphagnum* moss-dominated northern peatlands, which act as methane sinks and hence are important players in the global carbon cycle.

## Supplementary Files

Supplementary File 1
