# Supplementary material for: High Throughput Sequencing to Detect Differences in Methanotrophic Methylococcaceae and Methylocystaceae in Surface Peat, Forest Soil, and Sphagnum Moss in Cranesville Swamp Preserve, West Virginia, USA
Source: Microorganisms. 2015 Apr 2;3(2):113–36. doi: 10.3390/microorganisms3020113 (PMC5023241; doi:10.3390/microorganisms3020113)
Supplement: Supplementary File 1 [file microorganisms-03-00113-s001.docx]

**Supplementary Information**


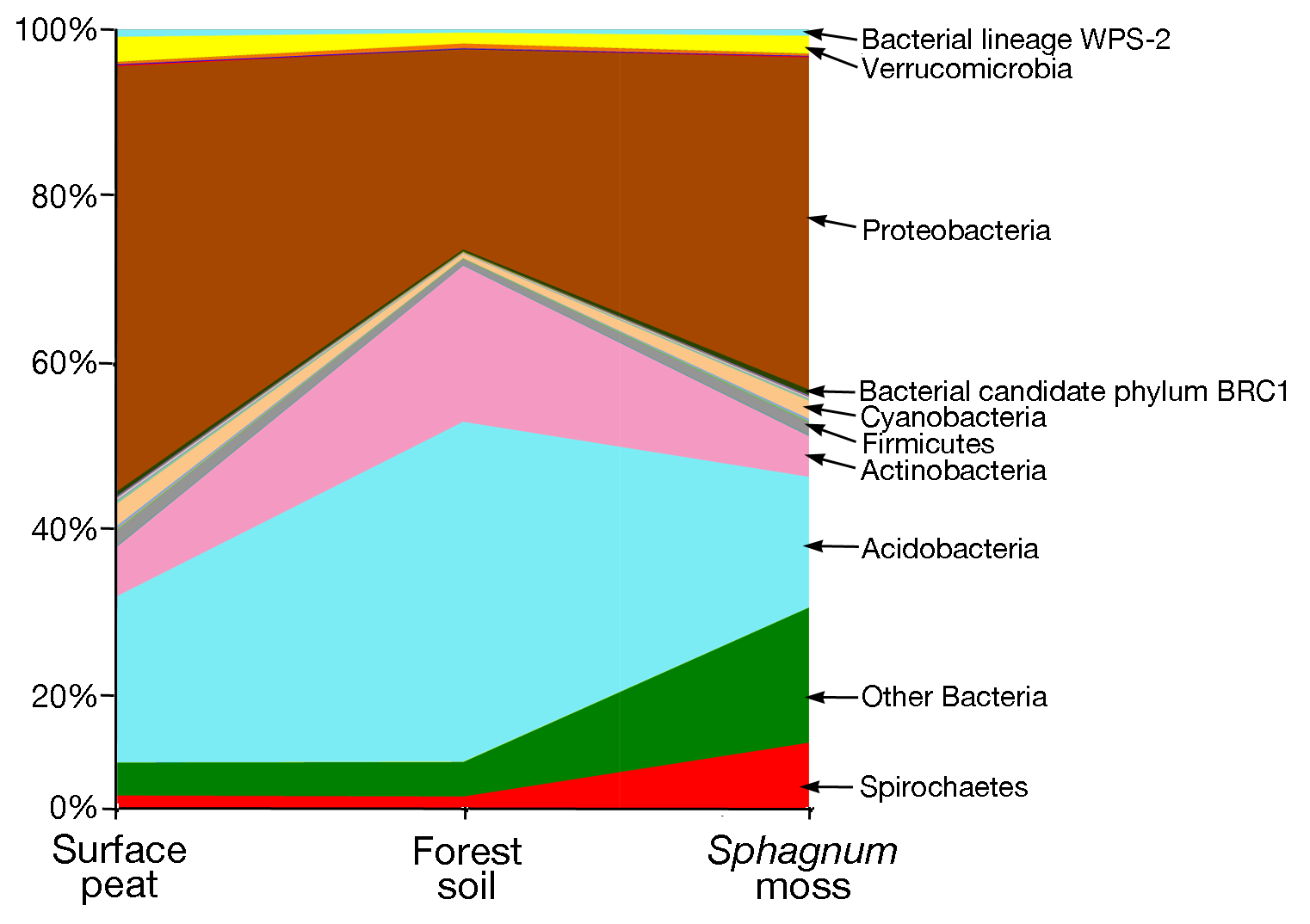


**Figure S1.** Relative sequence abundance of bacterial phyla and candidate divisions based on the 52,134 unique OTU reads between surface peat, forest soil and *Sphagnum* moss. Acidobacteria and Proteobacteria were detected consistently (at >20% of the total composition) at all 3 sites.

| 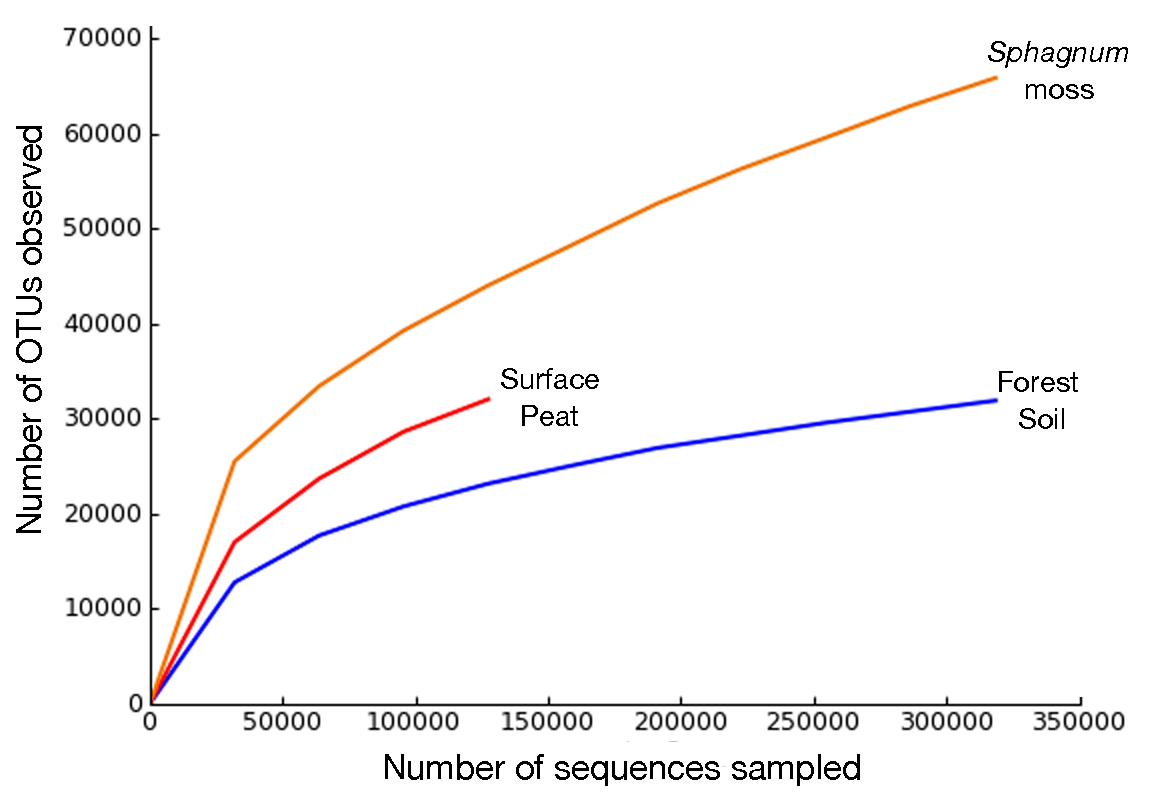 |
| --- |
| (**a**) |
| 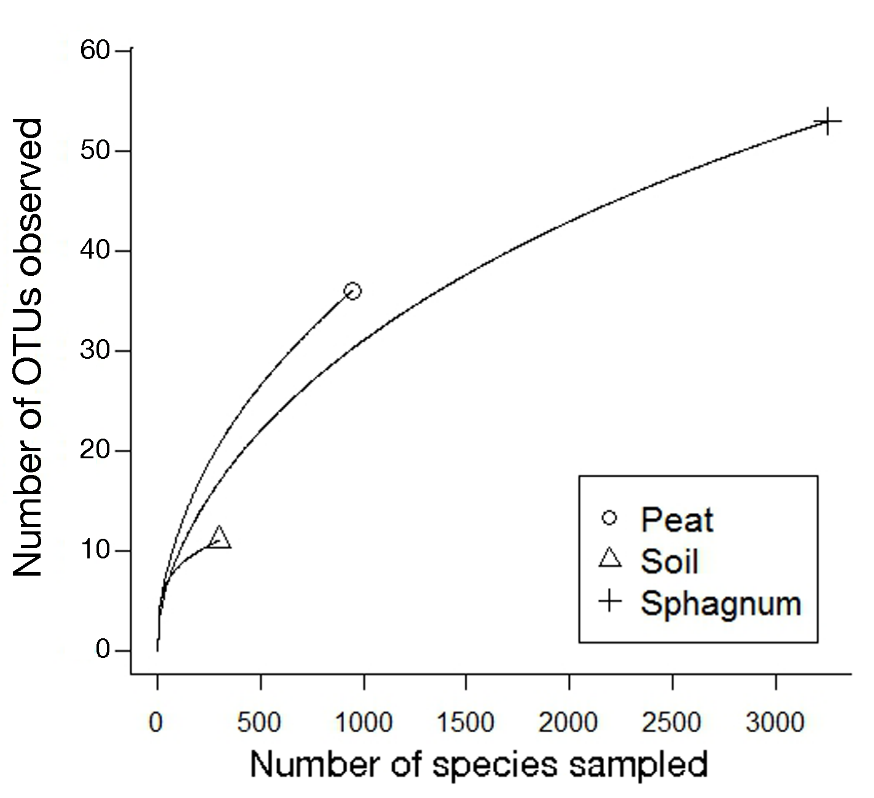 |
| (**b**) |

**Figure S2.** Bacterial taxon richness in surface peat, forest soil and *Sphagnum* moss, assessed by rarefaction curves for (**a**) bacterial sequences, and (**b**) proteobacterial methanotroph OTUs (from the Methylococcaceae and Methylocystaceae) in this study. The rarefaction curves show the relationship between increasing the number of random samplings and new, unique bacterial OTUs in order to assess species richness from the results of sampling.

**Figure S3.** Phylogenetic tree based on maximum parsimony (MP) analysis of 296 OTUs (~196 bp) related to the Methylacidiphilaceae detected in this study (in bold) in comparison with their close relatives. Bootstrap values from 1000 replicates are indicated at the nodes of branches (if > 50). The scale bar represents the number of nucleotide changes.

**Table S1.** 16S rRNA gene sequences of 24 OTUs and 34 OTUs, which are related to the Methylococcaceae and Methylocystaceae, respectively, their accession numbers and % nucleotide identity with their closest cultured relatives. The number of times these sequences were detected in peat, forest soil, and *Sphagnum* moss in Cranesville Swamp Nature Preserve are indicated.

| **OTU** | **GenBank Accession no.** | **Closest Relative (GenBank  Accession no.)** | **% Identity** | **Peat** | **Soil** | ***Sphagnum* Moss** |
| --- | --- | --- | --- | --- | --- | --- |
| 21463 | LN624864 | *Methylovulum miyakonense* strain HT12 (NR_112920) | 97% | 11 | 0 | 12 |
| 21475 | LN624865 | *Methylomonas* sp. R-45382 (FR_798967.1) | 98% | 1 | 0 | 1 |
| 21511 | LN624866 | *Methylomonas paludis* strain MG30 (NR_108887.1) | 97% | 13 | 1 | 20 |
| 21675 | LN624867 | *Methylomonas* sp. M5 (HM564016.1) | 98% | 4 | 0 | 3 |
| 21729 | LN624868 | *Methylomonas* sp. R-45382 (FR_798967.1) | 97% | 1 | 0 | 1 |
| 21763 | LN624869 | *Methylovulum miyakonense* strain HT12 (NR_112920) | 97% | 2 | 0 | 0 |
| 21869 | LN624870 | *Methylomonas rubra* strain NCIMB 11913 (NR_114588) | 96% | 1 | 0 | 3 |
| 21893 | LN624871 | *Methylomonas* sp. R-45382 (FR_798967.1) | 96% | 1 | 0 | 0 |
| 21903 | LN624872 | *Methylomonas* sp. R-45382 (FR_798967.1) | 97% | 5 | 0 | 11 |
| 21953 | LN624873 | *Methylomonas paludis* strain MG30 (NR_108887.1) | 98% | 5 | 1 | 4 |
| 22015 | LN624874 | *Methylomonas* sp. R-45382 (FR_798967.1) | 96% | 1 | 0 | 0 |
| 22185 | LN624875 | *Methylomarinum vadi* strain T2–1 (AB453958) | 96% | 5 | 0 | 5 |
| 22267 | LN624876 | *Methylomonas* sp. M5 (HM564016.1) | 98% | 1 | 0 | 0 |
| 22452 | LN649237 | *Methylomonas* sp. R-45382 (FR_798967.1) | 98% | 1 | 0 | 0 |
| 23273 | LN624877 | *Methylomonas* sp. M5 (HM564016.1) | 96% | 0 | 0 | 1 |
| 23419 | LN624878 | *Methylomonas* sp. M5 (HM564016.1) | 98% | 0 | 0 | 1 |
| 23516 | LN624879 | *Methylomonas paludis* strain MG30 (NR_108887.1) | 96% | 0 | 0 | 2 |
| 23624 | LN624880 | *Methylomonas* sp. R-45382 (FR_798967.1) | 99% | 0 | 0 | 3 |
| 23880 | LN624881 | *Methylomonas* sp. M5 (HM564016.1) | 95% | 0 | 0 | 1 |
| 24005 | LN624882 | *Methylomonas paludis* strain MG30 (NR_108887.1) | 97% | 0 | 0 | 4 |
| 24081 | LN624883 | *Methylomarinum vadi* strain T2–1 (AB453958) | 96% | 0 | 0 | 2 |
| 24210 | LN624884 | *Methylomonas* sp. R-45382 (FR_798967.1) | 95% | 0 | 0 | 1 |
| 24262 | LN624885 | *Methylomonas paludis* strain MG30 (NR_108887.1) | 99% | 0 | 0 | 1 |
| 24304 | LN624886 | *Methylovulum miyakonense* strain HT12 (NR_112920) | 97% | 1 | 0 | 0 |
| 24461 | LN624887 | *Methylomonas* sp. M5 (HM564016.1) | 98% | 1 | 0 | 0 |
| 24516 | LN624888 | *Methylomonas paludis* strain MG30 (NR_108887.1) | 96% | 1 | 0 | 0 |

**Table S1.** *Cont.*

| **OTU** | **GenBank Accession no.** | **Closest Relative (GenBank  Accession no.)** | **% Identity** | **Peat** | **Soil** | ***Sphagnum* Moss** |
| --- | --- | --- | --- | --- | --- | --- |
| 25129 | LN624889 | *Methylomonas paludis* strain MG30 (NR_108887.1) | 96% | 0 | 0 | 1 |
| 25856 | LN624890 | *Methylomonas paludis* strain MG30 (NR_108887.1) | 96% | 0 | 0 | 1 |
| 25981 | LN624891 | *Methylomonas* sp. R-45382 (FR_798967.1) | 96% | 0 | 0 | 1 |
| 34472 | LN624830 | *Methylocystis heyeri* strain H2 (NR_042531) and *Methylocystis heyeri* strain Sakb1 (AM285681.1) | 100% | 13 | 3 | 10 |
| 36913 | LN624831 | *Methylocystis heyeri* strain H2 (NR_042531) and *Methylocystis heyeri* strain Sakb1 (AM285681.1) | 100% | 282 | 46 | 215 |
| 36914 | LN624832 | Type II methanotrophic strain RS5A-Re (AB669144.1) | 100% | 147 | 8 | 83 |
| 37265 | LN624833 | Type II methanotrophic strain RS5A-Re (AB669144.1) | 99% | 2 | 0 | 1 |
| 37536 | LN624834 | *Methylocystis heyeri* strain H2 (NR_042531) and *Methylocystis heyeri* strain Sakb1 (AM285681.1) | 100% | 320 | 68 | 1122 |
| 37943 | LN624835 | Type II methanotrophic strain RS5A-Re (AB669144.1) | 99% | 99 | 125 | 1595 |
| 38057 | LN624836 | *Methylocystis heyeri* strain H2 (NR_042531) and *Methylocystis heyeri* strain Sakb1 (AM285681.1) | 100% | 2 | 3 | 20 |
| 38454 | LN624837 | Type II methanotrophic strain RS5A-Re (AB669144.1) | 100% | 1 | 17 | 18 |
| 39681 | LN624838 | *Methylocystis heyeri* strain H2 (NR_042531) and *Methylocystis heyeri* strain Sakb1 (AM285681.1) | 100% | 1 | 24 | 60 |
| 41161 | LN624839 | Type II methanotrophic strain RS5A-Re (AB669144.1) | 100% | 0 | 0 | 1 |
| 42147 | LN624840 | *Methylocystis* sp. strain SC2 (NR_074220.1) and *Methylocystis* sp.SB2 (GU734136.1) | 99% | 0 | 0 | 2 |
| 42626 | LN624841 | *Methylocystis* sp. strain SC2 (NR_074220.1) and *Methylocystis* sp.SB2 (GU734136.1) | 99% | 0 | 0 | 1 |
| 42716 | LN624842 | *Methylocystis* sp. strain SC2 (NR_074220.1) and *Methylocystis* sp.SB2 (GU734136.1) | 99% | 0 | 0 | 2 |
| 42777 | LN624843 | *Methylocystis* sp. strain SC2 (NR_074220.1) and Methylocystis sp.SB2 (GU734136.1) | 99% | 0 | 0 | 1 |
| 42828 | LN624844 | Type II methanotrophic strain RS5A-Re (AB669144.1) | 99% | 0 | 0 | 1 |

**Table S1.** *Cont.*

| **OTU** | **GenBank Accession no.** | **Closest Relative (GenBank  Accession no.)** | **% Identity** | **Peat** | **Soil** | ***Sphagnum* Moss** |
| --- | --- | --- | --- | --- | --- | --- |
| 42875 | LN624845 | *Methylocystis heyeri* strain H2 (NR_042531) and *Methylocystis heyeri* strain Sakb1 (AM285681.1) | 99% | 0 | 0 | 1 |
| 42900 | LN624846 | *Methylocystis* sp. strain SC2 (NR_074220.1) and *Methylocystis* sp.SB2 (GU734136.1) | 99% | 0 | 0 | 1 |
| 42996 | LN624847 | Type II methanotrophic strain RS5A-Re (AB669144.1) | 99% | 0 | 0 | 1 |
| 43075 | LN624848 | *Methylocystis* sp. strain SC2 (NR_074220.1) and *Methylocystis* sp.SB2 (GU734136.1) | 99% | 7 | 0 | 8 |
| 43411 | LN624849 | *Methylocystis* sp. strain SC2 (NR_074220.1) and *Methylocystis* sp.SB2 (GU734136.1) | 100% | 1 | 0 | 1 |
| 43648 | LN624851 | *Methylocystis* sp. strain SC2 (NR_074220.1) and *Methylocystis* sp.SB2 (GU734136.1) | 99% | 3 | 0 | 3 |
| 43660 | LN624851 | Type II methanotrophic strain RS5A-Re (AB669144.1) | 99% | 1 | 0 | 2 |
| 43677 | LN624852 | Type II methanotrophic strain RS5A-Re (AB669144.1) | 99% | 2 | 0 | 7 |
| 43702 | LN624853 | *Methylocysti*s sp. S284 (HE798551.1) | 100% | 1 | 0 | 1 |
| 44721 | LN624854 | *Methylocystis* sp. strain SC2 (NR_074220.1) and *Methylocystis* sp.SB2 (GU734136.1) | 99% | 0 | 0 | 3 |
| 44821 | LN624855 | *Methylocystis* sp. strain SC2 (NR_074220.1) and *Methylocystis* sp.SB2 (GU734136.1) | 100% | 0 | 0 | 2 |
| 45047 | LN624856 | Type II methanotrophic strain RS5A-Re (AB669144.1) | 99% | 0 | 0 | 4 |
| 45243 | LN624857 | *Methylocysti*s *bryophila* S284 (HE798551.1) | 99% | 0 | 0 | 1 |
| 45663 | LN624858 | *Methylosinus sporium* (M95665.1) | 100% | 1 | 0 | 2 |
| 46432 | LN624859 | Type II methanotrophic strain RS5A-Re (AB669144.1) | 97% | 3 | 1 | 0 |
| 48530 | LN624860 | *Methylosinus sporium* (M95665.1) | 100% | 0 | 0 | 2 |
| 48657 | LN624861 | Type II methanotrophic strain RS5A-Re (AB669144.1) | 100% | 0 | 0 | 5 |
| 48954 | LN624862 | *Methylocysti*s sp. S284 (HE798551.1) | 100% | 1 | 0 | 0 |
| 49302 | LN624863 | Type II methanotrophic strain RS5A-Re (AB669144.1) | 99% | 2 | 0 | 1 |

**Table S2.** 16S rRNA gene sequences of 24 OTUs, which are related to methanotrophic members of the family Beijerinckiaceae, their accession numbers and % nucleotide identity with their closest cultured relatives. The number of times these sequences were detected in peat, forest soil, and *Sphagnum* moss in Cranesville Swamp Nature Preserve are indicated.

| **OTU** | **GenBank Accession no.** | **Closest Methanotrophic Relative  (GenBank Accession no.)** | **% Identity** | **Peat** | **Soil** | ***Sphagnum* Moss** |
| --- | --- | --- | --- | --- | --- | --- |
| 36177 | LN624892 | *Beijerinckia indica* subsp. indica strain ATCC 9039 (NR_074269.1), Methylocella silvestris strain BL2 (NR_074237.1) | 100% | 79 | 54 | 85 |
| 36226 | LN624893 | *Methylorosula* sp. strain J1 (HF568990.1) | 98% | 35 | 15 | 34 |
| 36361 | LN624894 | *Methylovirgula ligni* strain BW872 | 98% | 22 | 40 | 7 |
| 36380 | LN624895 | *Beijerinckia indica* subsp. indica strain ATCC 9039 (NR_074269.1), *Methylocella silvestris* strain BL2 (NR_074237.1) | 100% | 48 | 35 | 138 |
| 37505 | LN624896 | *Beijerinckia mobilis* strain ATCC 35011 (NR_112220.1), *Beijerinckia mobilis* strain DSM 2326 (NR_042180.1) | 98% | 4 | 1 | 3 |
| 37538 | LN624897 | *Methylovirgula ligni* strain BW872, *Methylovirgula lign*i strain BW863 | 99% | 10 | 13 | 119 |
| 38920 | LN624898 | *Methylovirgula ligni* strain BW872, *Methylovirgula lign*i strain BW863 | 99% | 0 | 3 | 7 |
| 41021 | LN624899 | *Bosea thiooxidans* strain HC89 (HG794268.1) | 99% | 0 | 0 | 2 |
| 41388 | LN624900 | *Beijerinckia mobilis* strain ATCC 35011, *Beijerinckia mobilis* strain DSM 2326 | 98% | 0 | 0 | 1 |
| 41462 | LN624901 | *Beijerinckia indica* subsp. indica strain ATCC 9039 (NR_074269.1), *Methylocella silvestri*s strain BL2 (NR_074237.1) | 100% | 0 | 0 | 1 |
| 41951 | LN624902 | *Methylocapsa aurea* strain KYG (NR_116996.1) | 99% | 0 | 0 | 1 |
| 42098 | LN624903 | *Sphingomonas* sp. 30D12 (GU932978) | 98% | 0 | 0 | 3 |
| 43225 | LN624904 | *Beijerinckia indica* subsp. indica strain ATCC 9039 (NR_074269.1), *Methylocella silvestri*s strain BL2 (NR_074237.1) | 100% | 2 | 0 | 1 |
| 43589 | LN624905 | *Methylorosula* sp. strain J1 (HF568990.1) | 98% | 2 | 1 | 0 |
| 43631 | LN624906 | *Beijerinckia indica* subsp. indica strain ATCC 9039 (NR_074269.1), *Methylocella silvestri*s strain BL2 (NR_074237.1) | 100% | 1 | 0 | 0 |
| 44117 | LN624907 | *Methylocapsa aurea* strain KYG (NR_116996.1) | 98% | 0 | 0 | 9 |
| 44684 | LN624908 | *Beijerinckia indica* subsp. indica strain ATCC 9039 (NR_074269.1), *Methylocella silvestri*s strain BL2 (NR_074237.1) | 98% | 0 | 1 | 0 |

**Table S2.** *Cont.*

| **OTU** | **GenBank Accession no.** | **Closest Methanotrophic Relative  (GenBank Accession no.)** | **% Identity** | **Peat** | **Soil** | ***Sphagnum* Moss** |
| --- | --- | --- | --- | --- | --- | --- |
| 44805 | LN624909 | *Beijerinckia indica* strain ATCC 19361 (AB119199.1), *Methylocella tundrae* strain Ch1(AJ563929.1) | 98% | 0 | 0 | 1 |
| 45353 | LN624910 | *Beijerinckia indica* subsp. indica strain ATCC 9039 (NR_074269.1), *Methylocella silvestri*s strain BL2 (NR_074237.1) | 98% | 0 | 0 | 1 |
| 45362 | LN624911 | *Methylocapsa aurea* strain KYG (NR_116996.1) | 98% | 0 | 0 | 1 |
| 47963 | LN624912 | *Methylovirgula ligni* strain BW872, *Methylovirgula lign*i strain BW863 | 97% | 0 | 0 | 1 |
| 48498 | LN624913 | *Methylovirgula ligni* strain BW872, *Methylovirgula lign*i strain BW863 | 95% | 0 | 1 | 0 |
| 48502 | LN624914 | *Methylovirgula ligni* strain BW872, *Methylovirgula lign*i strain BW863 | 97% | 0 | 0 | 1 |
| 48832 | LN624915 | *Beijerinckia indica* subsp. indica strain ATCC 9039 (NR_074269.1), *Methylocella silvestri*s strain BL2 (NR_074237.1) | 96% | 0 | 0 | 1 |
